# Successful embolisation of a spontaneous diffuse subcapsular liver bleeding in a patient receiving rivaroxaban

**DOI:** 10.1186/s42155-025-00617-z

**Published:** 2025-12-13

**Authors:** Darragh K. Waters, Jack Alderson, Douglas Mulholland

**Affiliations:** Department of Radiology, Beaumont Hospital, Dublin 9, Ireland

**Keywords:** Spontaneous hepatic haemorrhage, Subcapsular liver haematoma, Pseudoaneurysm, Transarterial embolisation, Gelfoam embolisation, Rivaroxaban-related bleeding, Hepatic artery embolisation, Interventional radiology

## Abstract

Spontaneous hepatic haemorrhage is a rare and potentially fatal condition. This case describes a 78-year-old woman on rivaroxaban who presented with haemodynamic shock due to a spontaneous subcapsular liver haematoma with capsular rupture and pseudoaneurysm formation. Imaging revealed multiple abnormal vessels without a single bleeding point. Transarterial embolisation with Gelfoam was performed, achieving haemostasis without significant hepatic infarction. The patient remained stable post-procedure, with normalisation of liver function tests and no underlying liver neoplasm on follow-up imaging. This case underscores the importance of early diagnosis and multidisciplinary intervention. Temporary embolic agents such as Gelfoam offer effective haemostasis with lower risk of long-term hepatic injury in patients with diffuse microvascular disruption.

## Introduction

Spontaneous hepatic haemorrhage is an uncommon but potentially fatal condition, typically associated with underlying liver pathology, trauma, or anticoagulation. Subcapsular haematomas may progress to capsular rupture and haemoperitoneum, with high associated morbidity and mortality [1]. Prompt diagnosis and multidisciplinary management are essential to prevent further haemorrhage and organ compromise. This case highlights a rare presentation of spontaneous subcapsular liver haematoma with capsular rupture and pseudoaneurysm formation in a patient anticoagulated with rivaroxaban, successfully managed with transarterial embolisation.

## Case report

A 78-year-old woman presented to the emergency department with sudden-onset abdominal pain, distension, and nausea. There was no history of trauma. Her medical history was significant for atrial fibrillation, and she was anticoagulated with rivaroxaban. On arrival, she was tachycardic and hypotensive, with a systolic blood pressure of 60 mmHg. Her haemoglobin level had dropped to 6.4 g/dL, necessitating blood transfusion. Portal venous phase computed tomography (CT) of the abdomen and pelvis revealed a large subcapsular haematoma in segments 5/6 of the liver with rupture of the overlying capsule and associated haemoperitoneum. Triple-phase CT of the abdomen demonstrated multiple abnormal vessels underlying the haematoma, suspicious for active haemorrhage.

Following multidisciplinary input from general surgery, general medicine, and intensive care, the patient was transferred to interventional radiology for hepatic angiography and embolisation. Via a right common femoral artery approach, a 5 French 65 cm Terumo C2 GLIDECATH and a 150 cm 0.035″ TERUMO GLIDEWIRE were used to select the coeliac artery and advanced into the right hepatic artery. Hand injected digital subtraction angiography revealed bleeding spots along the capsular surface of the right hepatic lobe. Proximal embolisation of the right hepatic artery was performed to stasis using Gelfoam slurry which was handcut and mixed with 5 ml normal saline and 5 ml iodinated contrast. Post-embolisation angiogram was satisfactory with stasis in the right hepatic arteries and no residual perfusion of the abnormal vessels. A 6 Fr Angioseal closure device was used for haemostasis in the right common femoral artery.

**Fig. 1:**
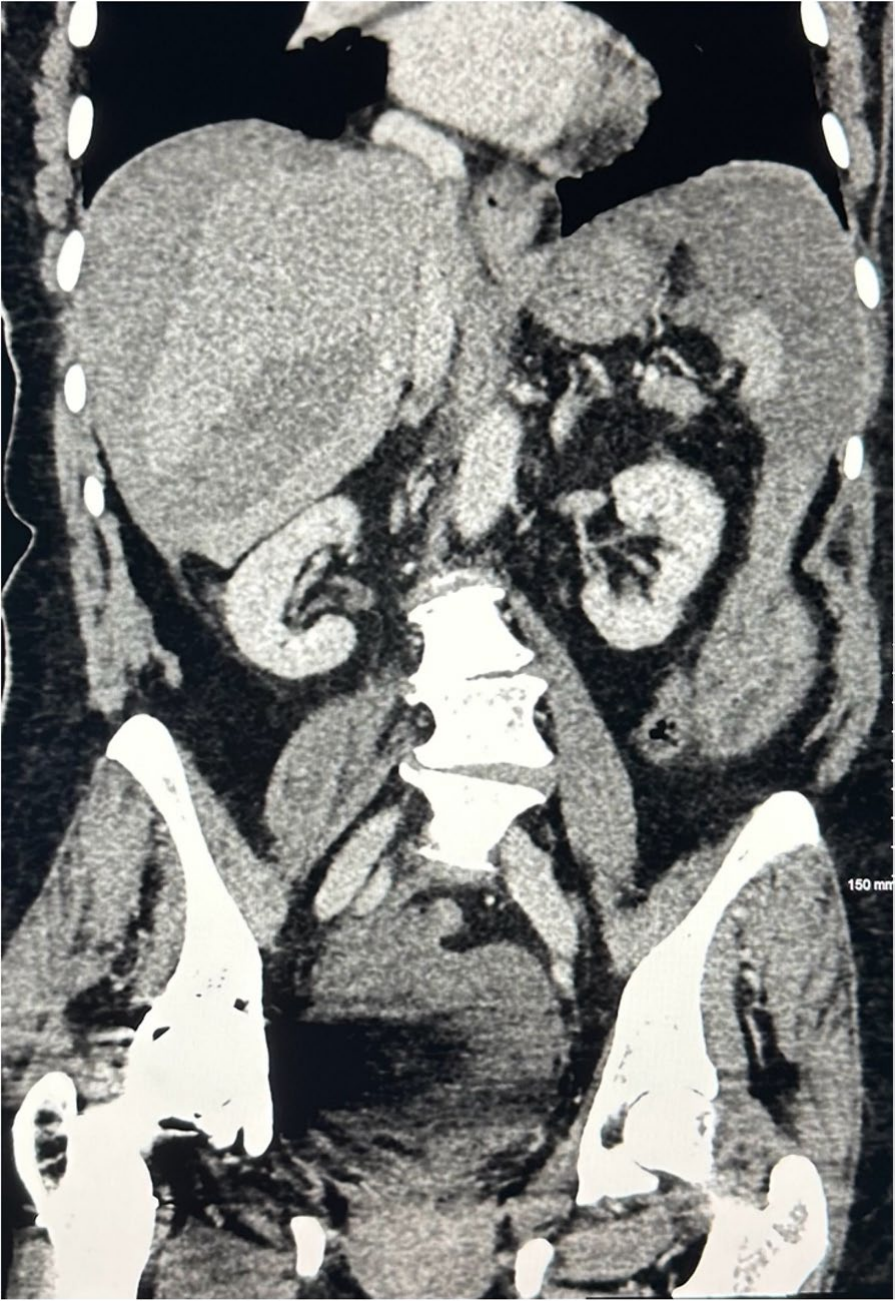
Coronal portal venous phase CT abdomen and pelvis showing hepatic laceration with ruptured capsule and subcapsular haematoma

**Fig. 2:**
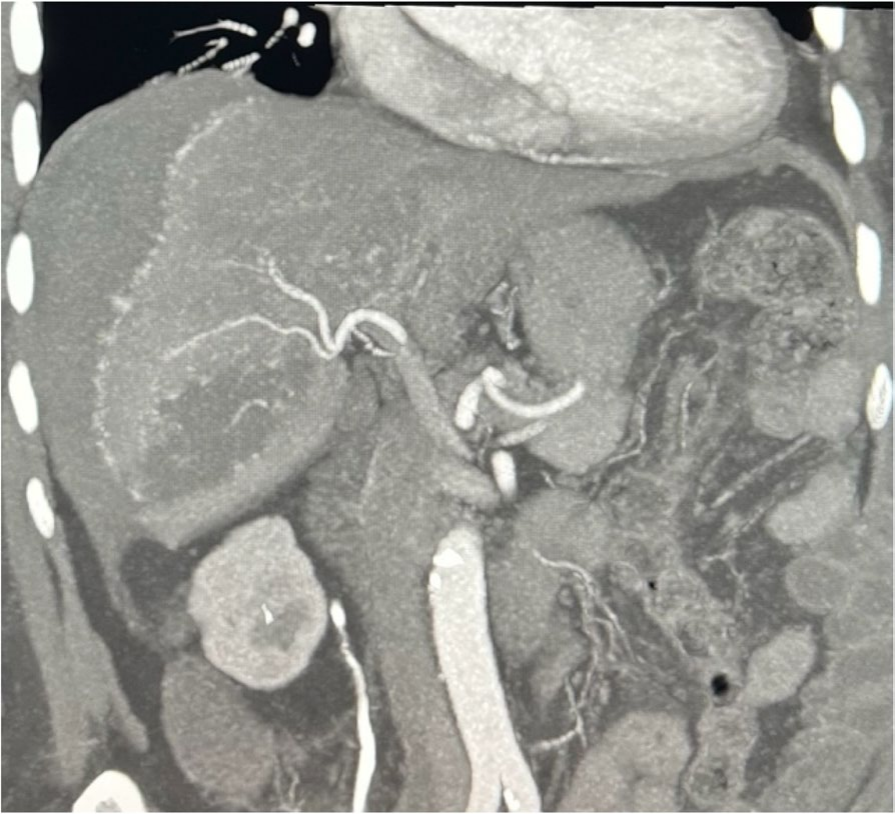
Coronal maximum intensity projection (MIP) arterial phase CT liver showing multiple abnormal vessels at the capsular surface of the right hepatic lobe

**Fig. 3:**
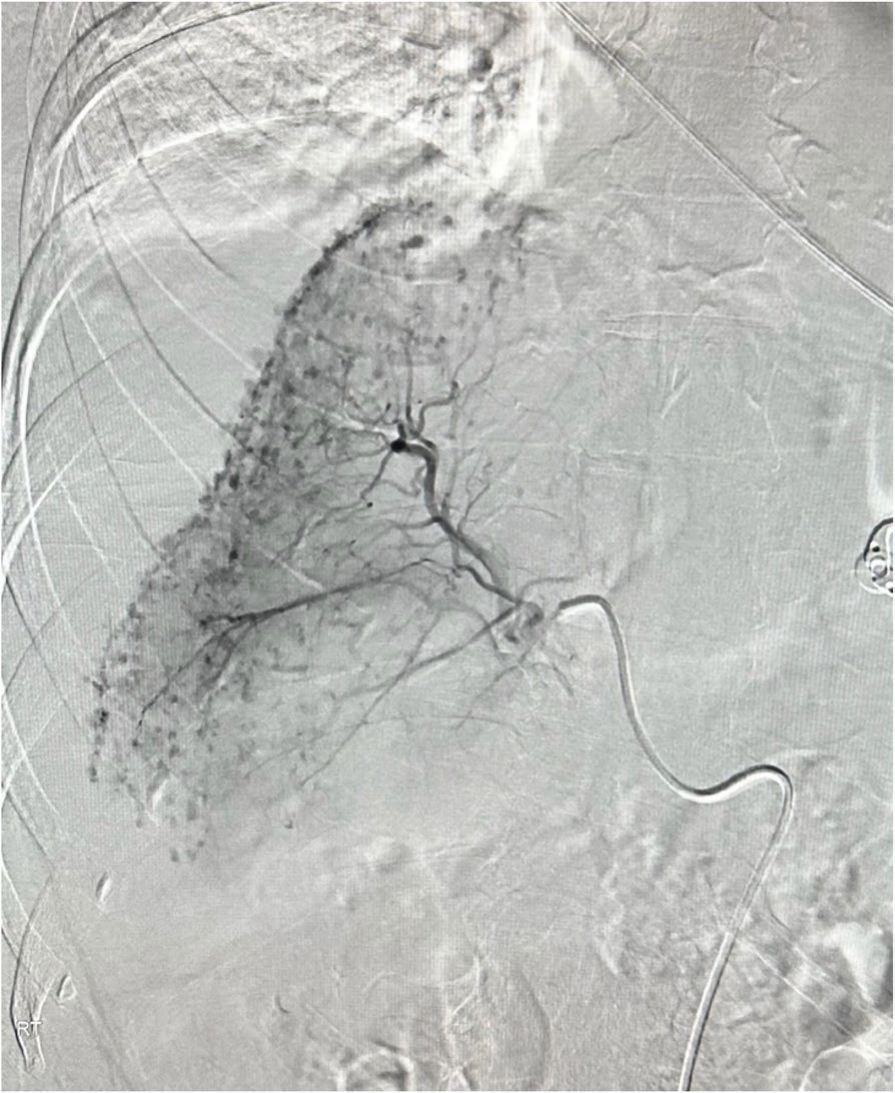
Right hepatic artery digital subtraction angiography (DSA) showing innumerable abnormal vessels overlying the entirety of the capsular surface of the right hepatic lobe

**Fig. 4:**
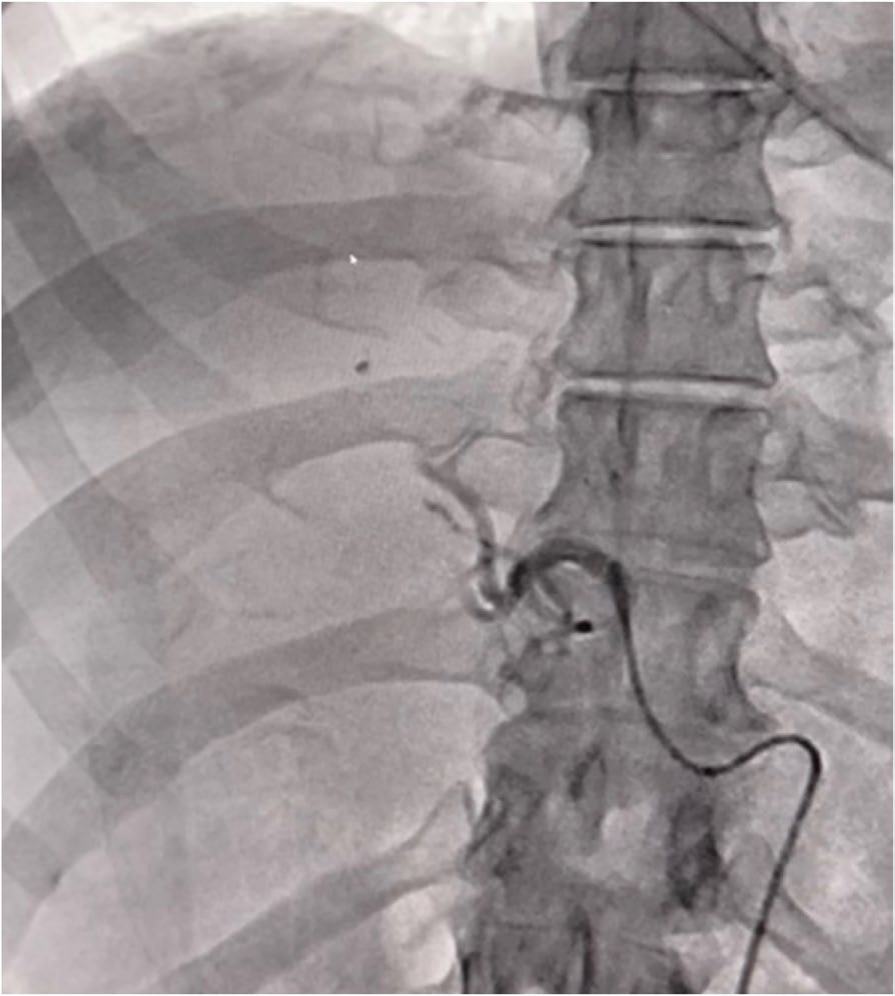
Right hepatic artery angiogram post-embolisation with Gelfoam showing stasis in the right hepatic arteries and no residual perfusion of the abnormal vessels

**Fig. 5:**
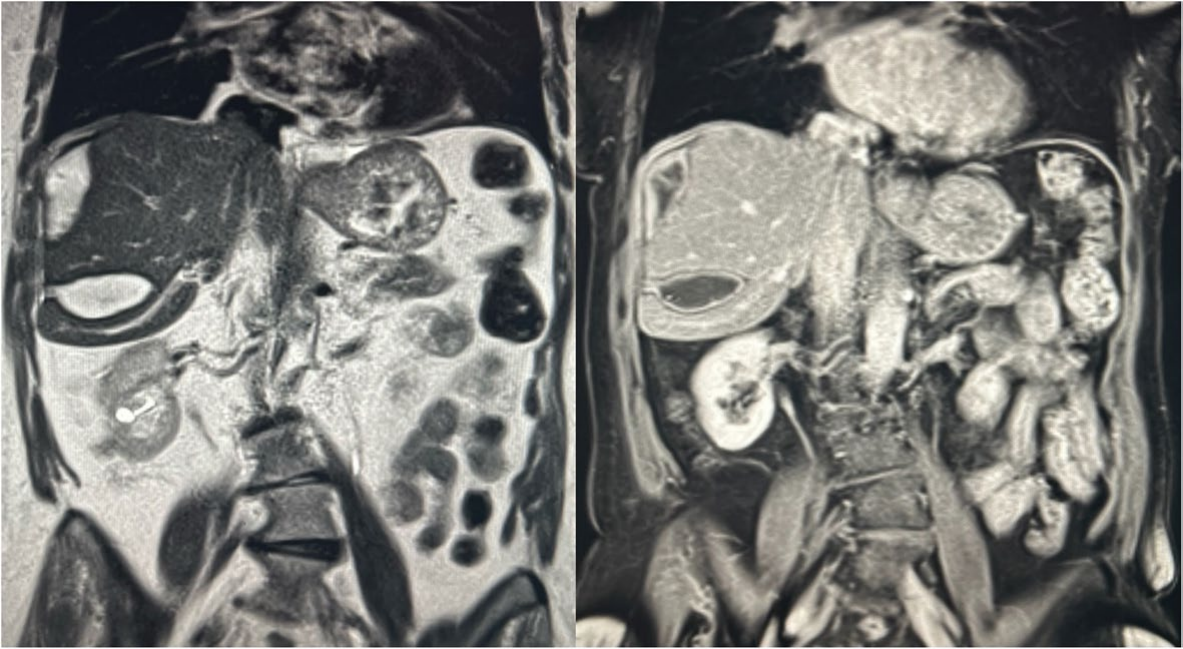
Coronal T2 weighted MRI image (left) and coronal T1 weighted MRI with contrast showing a reduction in haematoma size and no underlying liver lesions

Post-procedurally, the patient was transferred to the intensive care unit for monitoring. She remained haemodynamically stable and did not require further transfusion. Follow-up multiphase CT showed a stable haematoma with no residual perfusion of the abnormal vessels, though small areas of hepatic ischaemia were noted. Serial liver function tests post-procedure became mildly deranged with elevated alanine aminotransferase (ALT) and aspartate aminotransferase (AST). Bilirubin remained normal. Both ALT and AST normalised after 1 week. Elective outpatient magnetic resonance imaging (MRI) of the liver was performed 6 weeks post-procedure which showed reduction in size of the liver haematoma and no underlying liver lesions or infarction (Figs. 1, 2, 3, 4, and 5).

## Discussion

Spontaneous subcapsular haematoma of the liver is a rare and life-threatening condition, typically associated with underlying liver lesions or occurring in the context of HELLP syndrome (Hemolysis, Elevated Liver Enzymes, and Low Platelets) during pregnancy [2]. In the obstetric population, mortality from a ruptured subcapsular haematoma has been reported to range between 39 and 59% [1]. As the haematoma enlarges, shear forces may cause avulsion of the hepatic capsule from the underlying parenchyma, leading to tearing of intrahepatic or subcapsular arteries. This disruption in arterial wall integrity allows blood to escape into the surrounding tissue and form contained haematomas, known as pseudoaneurysms. Unlike true aneurysms, these lack all three arterial wall layers and are walled off by surrounding parenchyma or capsule. The resulting high-pressure arterial flow within these fragile structures increases the risk of ongoing or delayed haemorrhage. In this patient, the presence of innumerable pseudoaneurysms along the capsular surface of the right hepatic lobe likely reflects diffuse microvascular disruption secondary to subcapsular haematoma expansion and capsular rupture. Notably, the patient had a normal outpatient MRI 2 months prior to presentation, with no evidence of underlying liver disease or lesion. Spontaneous hepatic haematoma is exceedingly rare in patients without predisposing liver pathology. The estimated annual risk of spontaneous major haemorrhage while taking rivaroxaban is approximately 1%, only marginally higher than with aspirin [2].

Management options for acute liver haemorrhage include conservative measures, angiography with embolisation, and surgery. Hepatic artery embolisation is an effective treatment with one study demonstrating success rates of up to 94% [3]. In this case, the presence of diffuse haemorrhage without a single identifiable bleeding point made permanent embolic agents such as coils or glue less suitable. Instead, Gelfoam was selected due to its ability to achieve diffuse embolisation. The Gelfoam was prepared into a slurry mixed with normal saline and iodinated contrast. Gelfoam slurry is known to contain very small particles to which facilitates a more distal embolisation [4].

As an absorbable gelatin sponge, Gelfoam promotes mechanical occlusion and platelet aggregation, resulting in temporary cessation of blood flow. It is gradually resorbed over several weeks, allowing for revascularisation of the embolised tissue. In this case, Gelfoam was selected due to the diffuse distribution of haemorrhage in the right hepatic lobe, necessitating proximal embolisation from the right hepatic artery. Permanent occlusion of a major hepatic artery carries a higher risk of significant liver ischaemia, particularly in patients without robust collateral supply. The use of Gelfoam mitigates this risk by allowing temporary haemostasis while preserving the potential for eventual reperfusion, which is particularly beneficial in older patients or those with limited hepatic reserve. This was reflected in the follow-up imaging, which showed only small areas of hepatic infarction and transient liver function test (LFT) derangement.

Surveillance imaging is important to ensure no underlying liver lesions. Our literature search revealed a similar case of hepatic capsular avulsion following video-assisted thoracoscopic surgery (VATS) [5]. In this case, the patient became systemically unwell with deranged LFTs. Surveillance imaging revealed infarction of the right hepatic lobe. Our patient did have small areas of hepatic infarction and mildly deranged LFTs which normalised after 1 week.

## Conclusion

Spontaneous hepatic haemorrhage is a rare but life-threatening condition that requires rapid recognition and multidisciplinary management. This case demonstrates that transarterial embolisation with temporary agents such as Gelfoam can achieve effective haemostasis in the setting of diffuse pseudoaneurysm formation, while limiting the risk of long-term hepatic infarction. Careful follow-up is essential to exclude underlying hepatic pathology and to monitor recovery of liver function.

## Data Availability

Data sharing is not applicable to this article as no datasets were generated or analysed during the current study.

## Abbreviations

ALT: Alanine aminotransferase
AST: Aspartate aminotransferase
CT: Computed tomography
HELLP: Hemolysis, Elevated Liver Enzymes, and Low Platelets
ICU: Intensive care unit
LFT: Liver function test
MRI: Magnetic resonance imaging
VATS: Video-assisted thoracoscopic surgery
